# Somatic Point Mutations in mtDNA Control Region Are Influenced by Genetic Background and Associated with Healthy Aging: A GEHA Study

**DOI:** 10.1371/journal.pone.0013395

**Published:** 2010-10-14

**Authors:** Giuseppina Rose, Giuseppe Romeo, Serena Dato, Paolina Crocco, Amalia C. Bruni, Antti Hervonen, Kari Majamaa, Federica Sevini, Claudio Franceschi, Giuseppe Passarino, the GEHA Project Consortium

**Affiliations:** 1 Department of Cell Biology, University of Calabria, Rende, Italy; 2 Regional Center for NeuroGenetics, Lamezia Terme, Italy; 3 Laboratory of Gerontology, Tampere School of Public Health, Tampere, Finland; 4 Department of Neurology, University of Oulu, Oulu, Finland; 5 Interdepartmental Centre “Luigi Galvani” (CIG), University of Bologna, Bologna, Italy; Ben-Gurion University of the Negev, Israel

## Abstract

Tissue specific somatic mutations occurring in the mtDNA control region have been proposed to provide a survival advantage. Data on twins and on relatives of long-lived subjects suggested that the occurrence/accumulation of these mutations may be genetically influenced. To further investigate control region somatic heteroplasmy in the elderly, we analyzed the segment surrounding the nt 150 position (previously reported as specific of Leukocytes) in various types of leukocytes obtained from 195 ultra-nonagenarians sib-pairs of Italian or Finnish origin collected in the frame of the GEHA Project. We found a significant correlation of the mtDNA control region heteroplasmy between sibs, confirming a genetic influence on this phenomenon. Furthermore, many subjects showed heteroplasmy due to mutations different from the C150T transition. In these cases heteroplasmy was correlated within sibpairs in Finnish and northern Italian samples, but not in southern Italians. This suggested that the genetic contribution to control region mutations may be population specific. Finally, we observed a possible correlation between heteroplasmy and Hand Grip strength, one of the best markers of physical performance and of mortality risk in the elderly. Our study provides new evidence on the relevance of mtDNA somatic mutations in aging and longevity and confirms that the occurrence of specific point mutations in the mtDNA control region may represent a strategy for the age-related remodelling of organismal functions.

## Introduction

It has been recognized for a long time that age-related random damages to mtDNA and the consequent decrease in the respiratory chain capacity are among the major contributors to the aging process [1]–[4]. In fact, several studies reported that deletions and point mutations of mtDNA accumulate during aging in humans and in a wide range of organisms [5], [6]. However, in the last decade different studies highlighted specific somatic mutations in the mtDNA Control Region (CR) which can reach high levels in aged individuals [7], [8]. These mutations are tissue-specific and occur at mtDNA sites which are critical for replication or transcription, suggesting new important clues on the relevance of mtDNA heteroplasmic mutations in the aging process. For instance, the C150T transition that has been observed in leukocytes and fibroblasts creates a new replication site at position 149, substituting for that at 151.

Interestingly, it has been found that the CR heteroplasmic point mutations are over-represented in centenarians with respect to younger subjects in the Italian population. Data on MZ and DZ twin pairs have proposed that the heteroplasmic levels of 150C and 150T alleles were genetically controlled [9]. This hypothesis has been bolstered by analyzing centenarians' families, where we demonstrated that CR heteroplasmy in centenarians' descendants (children and nieces/nephews) are significantly higher than in age-matched controls and, moreover, they are significantly correlated in parent-offspring pairs [10]. Thus, it has been proposed that the CR somatic point mutations may represent a remodelling mechanism which would restore the replication machinery, providing a beneficial effect on longevity [9]. On the other hand, Iwata et al. [11], who analyzed the C150T mutation in leukocytes from centenarians and their offspring of an Ashkenazi Jew population, found a low incidence of the C150T mutation, but a rather high frequency of the T152C mutation. Furthermore, they found that the heteroplasmic form of the T152C transition (presumably originated from somatic events) increases with age. These findings clearly suggested population specificity on the occurrence of CR point mutations, possibly mediated by nuclear genetic variability. It may be worth noticing that population specificity has also been observed when the effect of inherited mtDNA variability on longevity has been studied [12], [13]. On the other hand no correlation was observed between inherited mtDNA variability and mtDNA CR heteroplasmy [10].

To get more insights about such complex phenomenon, in this study we took advantage of the population samples collected in the frame of the Genetics of Healthy Aging (GEHA) project. The GEHA consortium aimed to collect an unprecedented number of 90+ years old sib-pairs, from several European areas [14]. We analyzed mtDNA CR heteroplasmy in a 300 bp stretch surrounding the nt 150 position, in Lymphomonocytes and Granulocytes obtained from sib-pairs of different origin (Italy and Finland). This allowed us to further investigate the genetic control on the occurrence/accumulation of CR heteroplasmy, but also to analyze the cell specificity of CR somatic mutations. Moreover, we further analyzed the correlation between mtDNA polymorphisms and mtDNA CR heteroplasmy by defining for haplogroup classification the samples analyzed for heteroplasmy. Finally, due to the GEHA sampling strategy, we had an opportunity to verify a correlation between mtDNA heteroplasmy and health status in very old subjects (measured by means of Hand Grip strength, one the most reliable indicator of functional status in the elderly, see ref. [15] and references therein) as well as if population-specificity does exist for this phenomenon.

## Results

### MtDNA CR heteroplasmy

**Figure 1:**
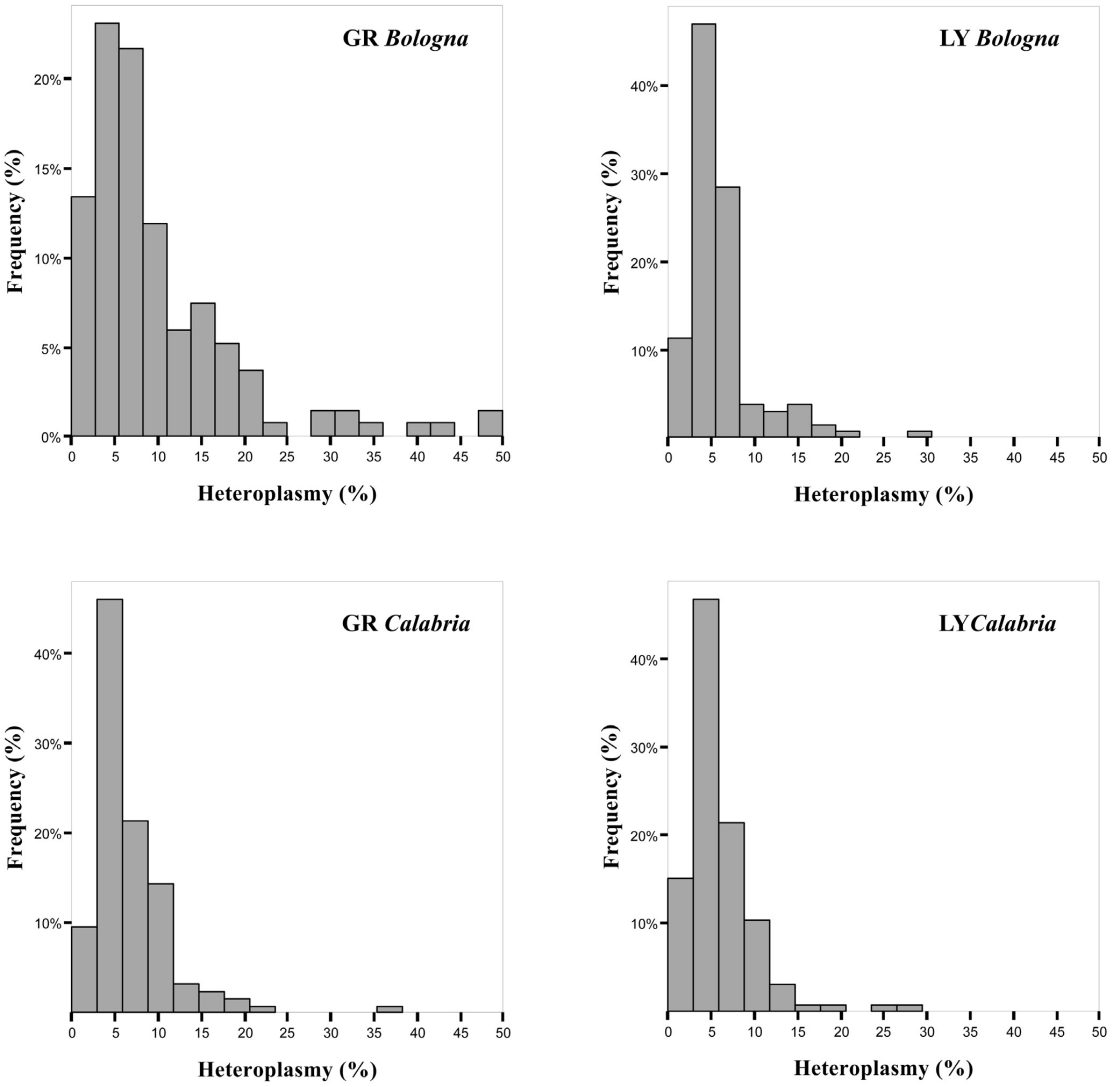
Heteroplasmy distribution in samples from northern and southern Italian sib-pairs. The histograms show the levels of heteroplasmy in Granulocytes (GR) and Lymphomonocytes (LY) of sib-pairs. The percentages of heteroplasmy are estimated on a DHPLC reference curve [10]. Northern Italian: Bologna; southern Italian: Calabria.

Fig.1 shows the distribution of heteroplasmy levels (as defined in Materials and Methods) in Granulocytes (GR) and Lymphomonocytes (LY) from subjects collected in Bologna and Calabria. In each sample the distribution is not normal, as the majority of the subjects showed levels of heteroplasmy lower than 10%.

**Figure 2:**
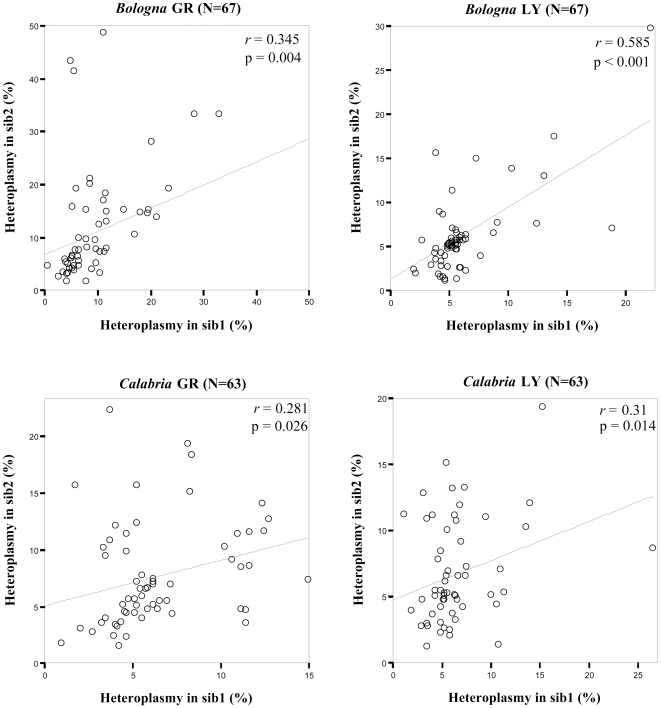
Correlation analyses in northern and southern Italian sib pairs. For each sample group regression lines and correlation coefficients (r) are shown. The p-values are obtained by 2-tailed Pearson test. Northern Italian: Bologna; southern Italian: Calabria.

Linear regression analyses showed a significant correlation in heteroplasmy between siblings in both cell types (Fig.2; p<0.05 in all the cases). Such a correlation was further confirmed by random permutation analysis, which showed that the correlation was lost when sib-pairs were shuffled. On the other hand, the distribution of heteroplasmy in GR and LY of each subject revealed a clear somatic contribution in the occurrence or accumulation of heteroplasmy. In fact, although a strong correlation of intra-individual heteroplasmy was observed (r = 0.9, p<0.001) some samples were found to be heteroplasmic in one cell type only.

**Figure 3:**
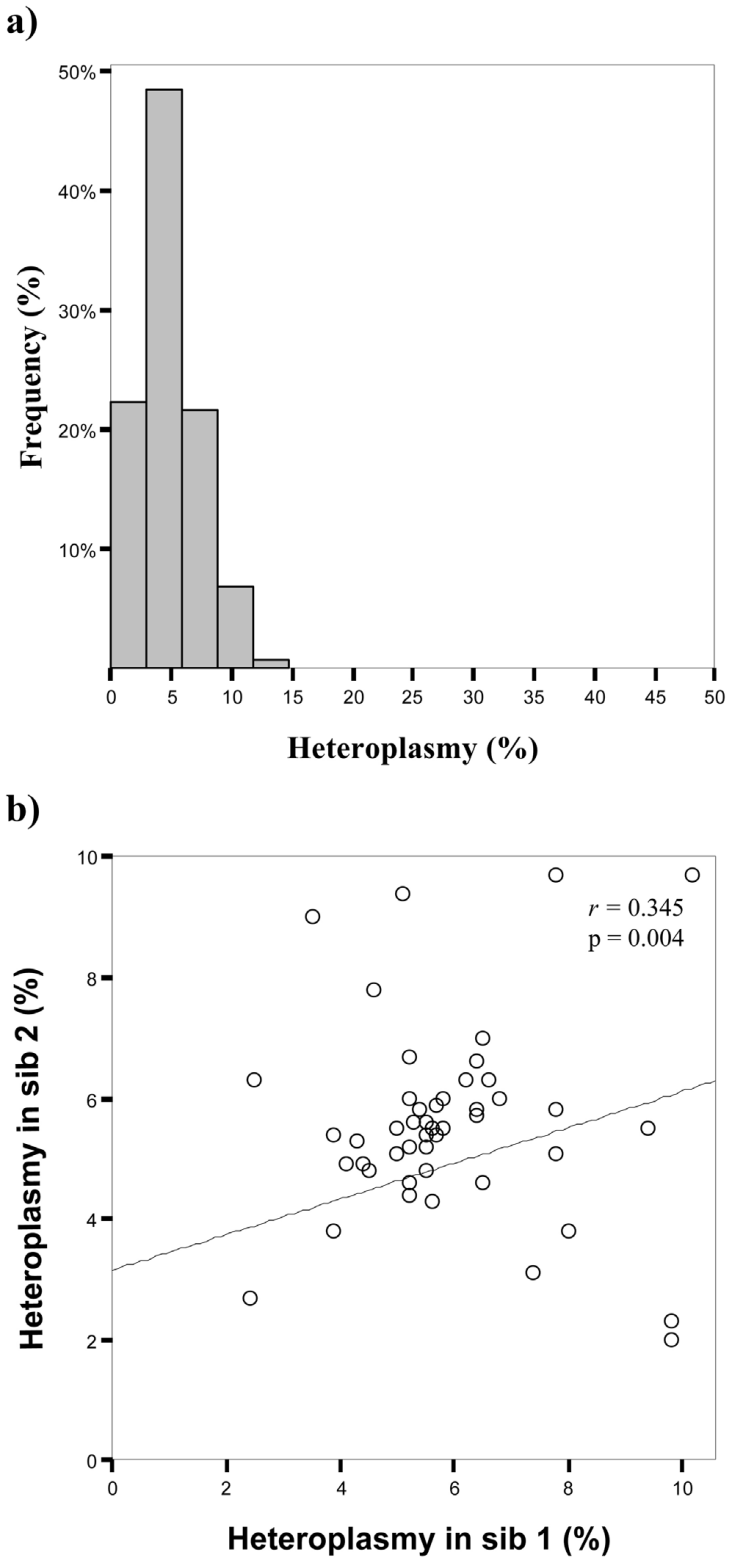
Heteroplasmy distribution (a) and correlation analyses (b) in Finnish sib-pairs. Percentages of heteroplasmy are estimated on a DHPLC reference curve [10]. In (b), regression analysis with correlation coefficients and p-values by 2-tailed Pearson test is shown.

We then studied the heteroplasmy in a group of sib-pairs from Finland (Tampere). For this sample, DNAs from buffy coats (BC) were available. Fig. 3a shows the distribution of mtDNA CR heteroplasmy levels. Also in this case we found a significant correlation between siblings (Fig. 3b) that was confirmed by random permutation analysis.

### Screening of the C150T mutation

**Figure 4:**
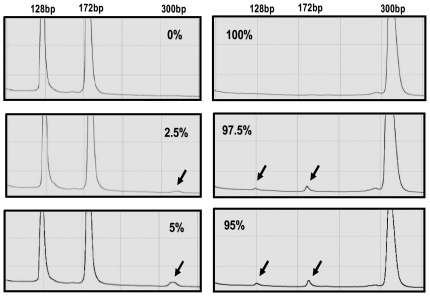
Chromatograms obtained for HPLC separation of digested samples with known heteroplasmy levels (PARFAH Method).

PARFAH method was used to specifically detect and quantify the heteroplasmic C150T mutation. In order to check the sensitivity of the PARFAH method, we first screened a series of samples with known levels of the heteroplasmic C150T mutation. We found that the method can reliably detect a mutant load as low as 2.5% (Fig. 4).

In Table 1 the results of the PARFAH and DHPLC analysis are summarized. We found that only a fraction of the heteroplasmic samples was heteroplasmic for the C150T mutation, while the majority of the samples were heteroplasmic for other point mutations.

**Table 1:** Proportion of samples heteroplasmic for the C150T mutation.

Sample group	Heteroplasmic samples (DHPLC-based analyses)	Samples heteroplasmic for the C150T transition (PARFAH-based analyses)
***Calabria*** ** GR**	124/126(98.4%)	25/124(20.2%)
***Calabria*** ** LY**	121/126(96%)	21/121(17.3%)
***Bologna*** ** GR**	121/134(90.3%)	29/121(24%)
***Bologna*** ** LY**	133/134(99.2%)	24/133(18%)
***Tampere*** ** BC**	108/130(83.1%)	30/108(27.8%)

**Figure 5:**
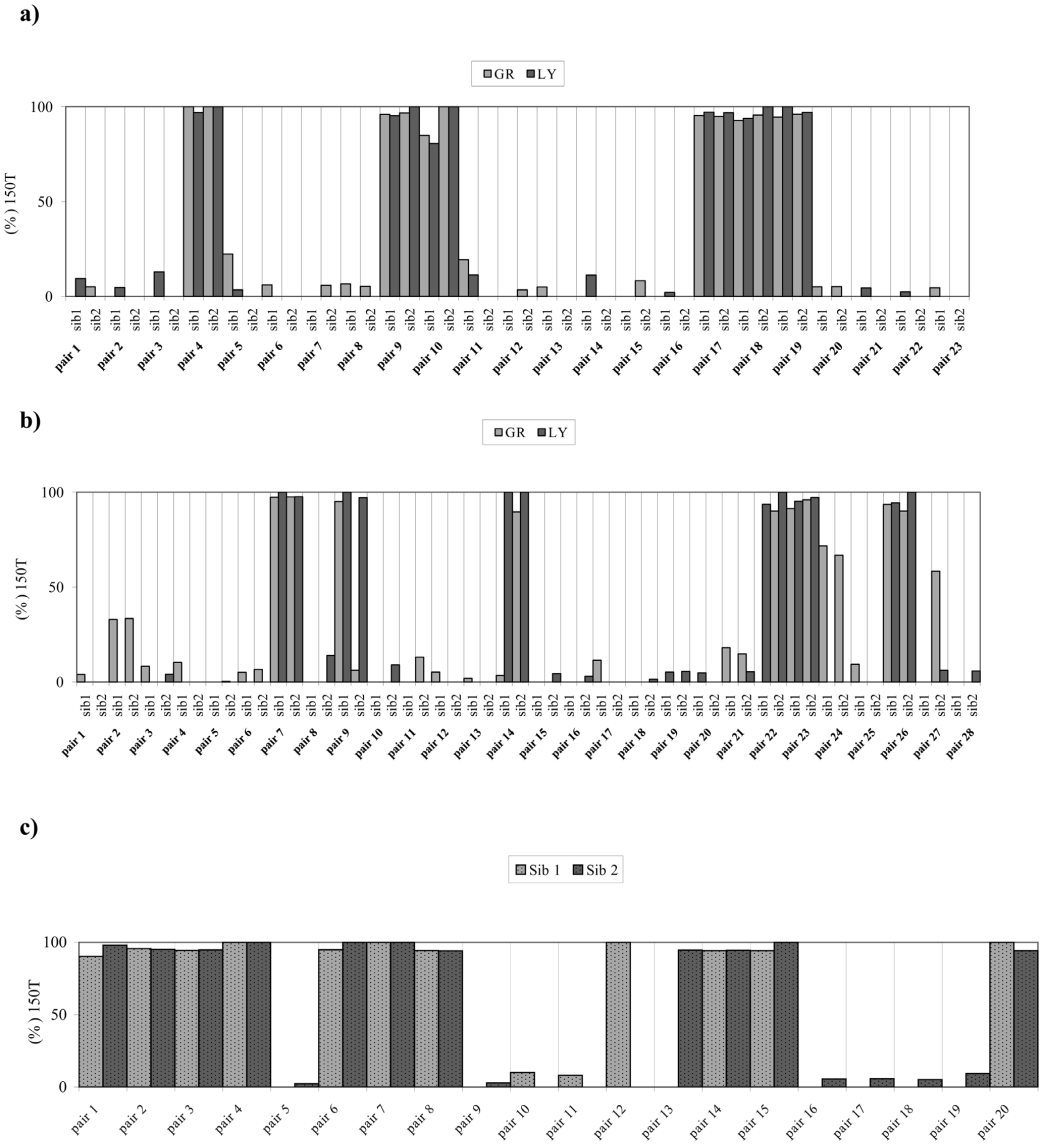
a) and b) Analysis of the C150T mutation in sib-pairs from *Calabria* and *Bologna* respectively. Bar graph summarizing the frequency and the distribution of the C150T in GR and LY. On the x-axis it is indicated each sib pair carrying the mutation; sib1 and sib2 refers respectively to the older and the younger sib in the pair. c) **Analysis of the C150T mutation in sib-pairs from Tampere**. Bar graph summarizing the frequency and the distribution of the C150T in Buffy Coats. On the x-axis each sib-pair carrying the mutation is indicated.

Fig. 5a and 5b show the levels of the 150T allele in both GR and LY of Calabria and Bologna sib-pairs. Only 23 out of 63 and 28 out of 67 sib-pairs, respectively, exhibited the mutation in one sib at least (either in GR or in LY). A very strong correlation between sibs of each pair was observed for both cell types (p<0.001 both in the Calabria and Bologna samples). Although a strong intra-individual correlation was observed (p<0.001 both in the Calabria and Bologna samples), some subjects were found to be heteroplasmic for the C150T mutation in one cell type only, indicating a somatic contribution in the occurrence or accumulation of the mutation.

Fig. 5c shows the levels of the 150T mutation in leukocytes of Finnish sib-pairs. Only 20 out of 65 sib-pairs exhibited the mutation at least in one sib. Also for the Finnish sib-pairs, a strong correlation in mutation levels was found (r = 0.885, p<0.001).

### Heteroplasmy due to mutations different than C150T

Table 1 shows that, by combining the data of DHPLC and PARFAH analyses, only a part of heteroplasmic samples was heteroplasmic for the C150T mutation. We then investigated whether the CR heteroplasmy not due to the C150T transition were correlated between sibs. Interestingly, a significant correlation was observed in Bologna and Tampere groups (p<0.05 in GR, LY and BC), but not in the Calabria group (p>0.05 in both GR and LY).

### Correlation of heteroplasmy with inherited mtDNA variability

Table S1 of Supplementary Information reports for each sample the levels of heteroplasmy and haplogroup classification according to Achilli et al [16] and Ghelli et al. [17]. No correlation was observed between haplogroup classification and mtDNA CR heteroplasmy.

### Correlation of heteroplasmy with physical performance

For this analysis, we considered only the elder sib from each pair. In addition, we pooled the Italian samples, for which we had data for both Lymphomonocytes and Granulocytes. We found a correlation between heteroplasmy in Lymphomonocytes and adjusted Hand Grip scores (r = 0.147, p = 0.037). Further, a marginal correlation was found (r = 0.165, p = 0.075) when we performed the same analysis on the heteroplasmy of the C150T transition. Finally, we observed a correlation (r = 0.223, p = 0.027) between the heteroplasmy in Lymphomonocytes not due to the C150T mutation and adjusted Hand Grip score. It may be worth noticing that if one uses the Bonferroni correction for multiple testing the significance threshold is 0.017, and then the observed correlations could not be considered significant.

No correlation whatsoever was observed between Hand Grip strength and the heteroplasmy level observed in the Granulocytes.

## Discussion

There is increasing evidence that tissue specific heteroplasmic mutations in the mtDNA CR may favor longevity. Moreover, the occurrence and the accumulation of these mutations may be genetically influenced [9], [10], [18]. In the current study we analyzed heteroplasmy in a segment of the mtDNA CR encompassing the nt 150 position. The analysis was carried out in Granulocytes and Lymphomonocytes from one individual in ultra-nonagenarians sib-pairs that were sampled from northern and southern Europe (Finns and Italians).

The strong correlation of the total CR heteroplasmy and the C150T heteroplasmy between sibs seems to confirm that CR heteroplasmy is genetically influenced. However, we found that CR heteroplasmy not related to C150T mutation is correlated between sib-pairs only in the Finnish and northern Italian samples but not in the southern Italian sample. These results suggest that the genetic contribution to CR mutations may be population specific. Thus, it is likely that nuclear genes, environmental factors or their interaction may have different effects on the occurrence or accumulation mtDNA somatic mutations. The analysis of heteroplasmy in GR and LY showed a high level of correlation, but also revealed some subjects with quite different heteroplasmy in the two cell types. This confirms that CR heteroplasmy is a somatic event, which occurs quite late in the differentiation of these two cell types with a common origin. Thus, the intra-individual correlation may be due to the genetic control of heteroplasmy, although we can not exclude the possibility that the somatic acquisition of heteroplasmy occurs in stem cells during haematopoiesis in most cases and after their differentiation only in a few cases.

The analysis of mtDNA haplogroups in our sample showed no correlation between CR mtDNA hetroplasmy and inherited mtDNA variability. This finding, in keeping with previous reports, confirms that the genetic factors affecting CR mtDNA hetroplasmy are likely to be nuclear genetic factors [9], [10]. In addition, as somatic mutations occurring in mtDNA CR fall on different mtDNA background given by a combination of polymorphisms described by haplogroup classification, our analysis suggests that the effect CR mtDNA mutation is not modified by mtDNA variability.

The correlation between Hand Grip strength and CR heteroplasmy in Lymphomonocytes suggested by our data is quite interesting although it will need to be verified in a larger sample. If confirmed, this data will further support the positive effect of CR point somatic mutations on longevity. In fact, Hand Grip strength is one of the best markers of health status in the elderly and it has been shown to be the best single survival predictor [15], [19]. It is important to point out that such a correlation was observed for Lymphomonocytes but not in the Granulocytes. In fact, we have previously proposed that the coexistence of mtDNA molecules carrying alternative replication origins due to specific CR mutations may represent an advantage in the frame of mitochondrial fusion and fission that regulates the remodeling of the mitochondrial network [20]. In fact, all the CR mutations that have been described [10], [11] were related to replication origins [21]. This strategy for restoring the functionality of damaged mitochondria that have accumulated during aging is crucial for maintaining the bioenergetic efficiency of the cell and seems to play an important role in cell differentiation [22], in the regulation of apoptotic events [23] and aging [24], [25]. Thus, it is evident that the presence of alternative replication origins may be more important for Lymphomonocytes, which have an important role in response to stress and are related to frailty [26], [27], than in Granulocytes, post mitotic cells living only for a few hours.

On the whole, our study has provided new important clues on the relevance of mtDNA somatic mutations, and of the consequent heteroplasmy, for aging and longevity. We confirmed the high incidence of somatic point mutations and the consequent heteroplasmy in the mtDNA CR of long-lived subjects. In addition, we showed that CR heteroplasmy is significantly correlated with physical performance in oldest olds. On the other hand, we showed that mtDNA heteroplasmy in Leukocytes may be due to the previously described C150T mutation, but also to other mutations, the occurrence of which may be influenced in a population-specific way. In any case, the genetic control of the mtDNA CR heteroplasmy suggests that the occurrence of mtDNA somatic mutations may represent an important strategy for the age-related remodeling of organismal functions.

## Materials and Methods

### Ethical statement

The sampling was carried out in the frame of the GEHA project [14], according to the directions of the Ethical Board of the GEHA Project, after receiving the approval of the local Ethical committees (in particular: Ethical Committee of the University of Calabria for the samples collected in Calabria; Independent Ethical Committee of the Bologna Hospital-University for the samples collected in Bologna; Ethical Committee of the city of Tampere for the samples collected in Finland). Written informed consent was obtained from each subject.

### Population samples

A total of 195 sib-pairs (390 subjects) were analyzed: 63 sib-pairs (126 subjects) were recruited in the south of Italy (Calabria), 67 sib-pairs (134 subjects) in the north of Italy (Bologna) and 65 sib-pairs (130 subjects) in Finland (Tampere). For the Italian sib-pairs we analyzed DNAs coming from two different cell-types, Granulocytes (GR) and Lymphomonocytes (LY), for a total of 520 DNA samples (252 from the south of Italy, 268 from the north of Italy); for the Finnish sib-pairs we analyzed 130 DNAs coming from Buffy Coats (BC).

### Biological samples

According to the standard operating procedure the biological samples (buffy coats, Lymphomonocytes and Granulocytes) were stored locally and shipped to the National Public Health Institute (Helsinki, Finland), where DNA extraction, quality control and storage of the extracted DNA were performed. An aliquot of these DNAs was sent to us for the genetic analysis.

### Quantification of the CR heteroplasmy by DHPLC

The mtDNA region under study (nt 16531-00261; 300 bp) was amplified and submitted to DHPLC [10]. The level of heteroplasmy, that is the percentage of the less frequent allele in the heteroplasmic mixture, was calculated as described by Rose et al. [10].

### Quantification of the C150T mutation levels by PARFAH

Quantification of the C150T mutation was carried out by PARFAH (PCR Amplicon Restriction Fragment Analysis by HPLC) [28]. About 600–800 ng (30 µl) of PCR products were digested with 10 U of FokI endonuclease (Biolabs) at 37°C for 3 hours by adding directly the enzyme into the PCR mix. 20 µl of digested products were injected into a DNASep™ column of a Transgenomic Wave Nucleic Acid Fragment Analysis System (Transgenomic, San Jose, CA) and eluted in 0.1 M triethylammonium acetate, pH 7, with a linear acetonitrile gradient at a flow rate of 0.9 ml/min under non-denaturing conditions (over temperature 50°C). By applying this method digested fragments eluted depending on their molecular weight. FokI cleaves amplified fragments carrying the 150C allele, but does not cleave amplified fragments carrying the 150T allele. Then, the presence of the homoplasmic 150C allele was recognised by the appearance of two peaks (relevant to the fragments of 128 and 172 bp); the presence of the homoplasmic 150T allele was recognised by the appearance of one peak (relevant to the fragments of 300 bp); the presence of heteroplasmic samples was recognised by the appearance of three peaks (relevant to the fragments of 128, 172 and 300 bp) in the elution profiles. In such a case, the percentage of mutant mtDNA was calculated by measuring the percentage of peak area (WAMAKER 4.0 software, Transgenomic San Jose) related to the mutant allele.

In order to check the sensitivity of the PARFAH method, we analyzed, in three independent experiments, a series of standard mixes in which the percentage of mutant mtDNA varied from 0 to 100%.

### Analysis of mtDNA inherited variability

MtDNA variability was analyzed by defining subhaplogroup of one subject of each sib pair. The analysis was carried out by sequencing the entire mtDNA control region from nucleotide position (np) 16024 to np 576. This was followed by a hierarchical survey of haplogroup and sub-haplogroup diagnostic markers in the coding region [16], [17].

### Physical performance

Physical performance was evaluated by means of Hand grip strength. Hand grip strength was measured by using a handheld dynamometer while the subject was sitting with the arm close to his/her body. The test was repeated three times with the stronger hand; the maximum of these values was used in the analyses. The scores obtained were subsequently adjusted for sex, age and height as these parameters turned out to be significantly correlated with Hand Grip strength.

### Statistical analyses

The two-tailed Pearson test was used to perform correlation analyses. The linear correlation coefficient (*r*) measures the strength and the direction of a linear relationship between two variables; while the p-value measures the probability of getting a correlation as large as the observed value by random chance, when the true correlation is zero. The p-value is computed by transforming the correlation to create a *t* statistic having n-2 degrees of freedom, where *n* is the number of data pairs.

Permutation procedures were used to verify if the observed correlation in mtDNA CR heteroplasmy in sib-pairs was due to the kinship between siblings or to their concordant age.

All statistical analyses were performed by using SPSS 14.0 software (SPSS Inc., Chicago, IL, USA). A significance level of α = 0.05 was chosen in all the tests.

## Supporting Information

Table S1 Phylogenetic classification of the samples analyzed according to the haplogroup classification in comparison with the levels of heteroplasmy.
(0.65 MB DOC)
