# Supplementary material for: Somatic Point Mutations in mtDNA Control Region Are Influenced by Genetic Background and Associated with Healthy Aging: A GEHA Study
Source: PLoS One. 2010 Oct 14;5(10):e13395. doi: 10.1371/journal.pone.0013395 (PMC2954809; doi:10.1371/journal.pone.0013395)
Supplement: Table S1 — Phylogenetic classification of the samples analyzed according to the haplogroup classification in comparison with the levels of heteroplasmy. (0.65 MB DOC) [file pone.0013395.s001.doc]

**Table S1**. Phylogenetic classification of the samples analyzed according to the haplogroup classification [see ref 16 and 17] in comparison with the levels of heteroplasmy.

For each sib pair only one subject (indicated as A) was analyzed. The classification of the second sib (B) was inferred and it is reported in italics.

% HetLY indicates the percentage of heteroplasmy in Lymphomonocytes. % HetGR indicates the percentage of heteroplasmy in Granulocytes. % HetBC indicates the percentage of heteroplasmy in Buffy Coats.

| **Sib Pairs** | **Recruitment Center** | **% Het LY** | **% Het GR** | **% Het BC** | **Phylogenetic Classification** |
| --- | --- | --- | --- | --- | --- |
| 1-A | Bologna | 4,33 | 3,88 |  | HV0* |
| 1-B | Bologna | 4,25 | 5,53 |  | *HV0** |
| 2-A | Bologna | 22,08 | 32,87 |  | I |
| 2-B | Bologna | 29,76 | 33,39 |  | *I* |
| 3-A | Bologna | 5,53 | 5,60 |  | H1 |
| 3-B | Bologna | 5,83 | 7,69 |  | *H1* |
| 4-A | Bologna | 7,61 | 7,92 |  | X2c |
| 4-B | Bologna | 3,96 | 8,24 |  | *X2c* |
| 5-A | Bologna | 5,60 | 10,33 |  | H* |
| 5-B | Bologna | 6,60 | 7,37 |  | *H** |
| 6-A | Bologna | 3,66 | 0,35 |  | H* |
| 6-B | Bologna | 4,33 | 4,70 |  | *H** |
| 7-A | Bologna | 6,06 | 5,08 |  | H1 |
| 7-B | Bologna | 5,83 | 6,60 |  | *H2* |
| 8-A | Bologna | 4,85 | 5,53 |  | T2 |
| 8-B | Bologna | 5,00 | 4,77 |  | *T2* |
| 9-A | Bologna | 1,91 | 2,49 |  | U5b2 |
| 9-B | Bologna | 2,42 | 2,64 |  | *U5b2* |
| 10-A | Bologna | 10,33 | 19,42 |  | T2 |
| 10-B | Bologna | 13,95 | 14,74 |  | *T2* |
| 11-A | Bologna | 5,83 | 6,21 |  | H* |
| 11-B | Bologna | 6,14 | 6,44 |  | *H** |
| 12-A | Bologna | 4,25 | 4,92 |  | T2b |
| 12-B | Bologna | 2,93 | 6,14 |  | *T2b* |
| 13-A | Bologna | 5,98 | 7,69 |  | H3 |
| 13-B | Bologna | 6,29 | 9,76 |  | *H3* |
| 14-A | Bologna | 5,45 | 6,29 |  | H* |
| 14-B | Bologna | 5,15 | 5,60 |  | *H** |
| 15-A | Bologna | 9,03 | 11,23 |  | X2b |
| 15-B | Bologna | 7,76 | 7,37 |  | *X2b* |
| 16-A | Bologna | 13,95 | 19,52 |  | H1b |
| 16-B | Bologna | 17,53 | 15,36 |  | *H1b* |
| 17-A | Bologna | 0,00 | 11,56 |  | HV0* |
| 17-B | Bologna | 14,82 | 13,09 |  | *HV0** |
| 18-A | Bologna | 5,00 | 6,37 |  | U7 |
| 18-B | Bologna | 5,53 | 4,77 |  | *U7* |
| 19-A | Bologna | 7,22 | 20,10 |  | H1c |
| 19-B | Bologna | 15,00 | 28,23 |  | *H1c* |
| 20-A | Bologna | 5,53 | 5,23 |  | H5 |
| 20-B | Bologna | 6,91 | 6,37 |  | *H5* |
| 21-A | Bologna | 3,81 | 5,23 |  | H11 |
| 21-B | Bologna | 15,71 | 15,80 |  | *H11* |
| 22-A | Bologna | 4,25 | 5,38 |  | H* |
| 22-B | Bologna | 4,33 | 3,81 |  | *H** |
| 23-A | Bologna | 3,81 | 4,03 |  | H5 |
| 23-B | Bologna | 4,77 | 1,84 |  | *H5* |
| 24-A | Bologna | 2,56 | 10,41 |  | U5b |
| 24-B | Bologna | 5,76 | 3,37 |  | *U5b* |
| 25-A | Bologna | 4,40 | 4,18 |  | HV0* |
| 25-B | Bologna | 1,63 | 5,08 |  | *HV0** |
| 26-A | Bologna | 18,85 | 11,15 |  | H* |
| 26-B | Bologna | 7,06 | 17,07 |  | *H** |
| 27-A | Bologna | 3,96 | 4,03 |  | H* |
| 27-B | Bologna | 1,91 | 3,22 |  | *H** |
| 28-A | Bologna | 4,10 | 0,00 |  | H* |
| 28-B | Bologna | 9,03 | 13,17 |  | *H** |
| 29-A | Bologna | 3,44 | 3,44 |  | J1c |
| 29-B | Bologna | 3,00 | 3,59 |  | *J1c* |
| 30-A | Bologna | 4,63 | 4,55 |  | J1c1 |
| 30-B | Bologna | 4,03 | 4,33 |  | *J1c2* |
| 31-A | Bologna | 4,40 | 5,08 |  | HV* |
| 31-B | Bologna | 8,71 | 4,33 |  | *HV** |
| 32-A | Bologna | 5,23 | 0,00 |  | H* |
| 32-B | Bologna | 5,08 | 6,29 |  | *H** |
| 33-A | Bologna | 2,06 | 11,48 |  | K1c |
| 33-B | Bologna | 1,99 | 8,00 |  | *K1c* |
| 34-A | Bologna | 4,63 | 13,95 |  | H* |
| 34-B | Bologna | 1,41 | 0,00 |  | *H** |
| 35-A | Bologna | 5,15 | 7,76 |  | R0a |
| 35-B | Bologna | 5,53 | 1,77 |  | *R0a* |
| 36-A | Bologna | 4,77 | 21,08 |  | H1 |
| 36-B | Bologna | 4,77 | 13,95 |  | *H1* |
| 37-A | Bologna | 5,23 | 8,39 |  | H1 |
| 37-B | Bologna | 11,40 | 20,10 |  | *H1* |
| 38-A | Bologna | 4,92 | 18,00 |  | H* |
| 38-B | Bologna | 5,38 | 14,82 |  | *H** |
| 39-A | Bologna | 6,37 | 0,00 |  | HV0* |
| 39-B | Bologna | 2,27 | 9,92 |  | *HV0** |
| 40-A | Bologna | 12,40 | 11,40 |  | H* |
| 40-B | Bologna | 7,69 | 18,38 |  | *H** |
| 41-A | Bologna | 4,77 | 8,71 |  | T2b |
| 41-B | Bologna | 2,78 | 3,96 |  | *T2b* |
| 42-A | Bologna | 5,91 | 16,98 |  | H6b |
| 42-B | Bologna | 2,71 | 10,57 |  | *H6b* |
| 43-A | Bologna | 5,60 | 50,00 |  | H* |
| 43-B | Bologna | 4,70 | 6,98 |  | *H** |
| 44-A | Bologna | 5,76 | 23,41 |  | H6b |
| 44-B | Bologna | 6,06 | 19,42 |  | *H6b* |
| 45-A | Bologna | 5,60 | 5,91 |  | H1 |
| 45-B | Bologna | 1,41 | 19,42 |  | *H1* |
| 46-A | Bologna | 5,23 | 14,82 |  | HV* |
| 46-B | Bologna | 5,68 | 15,36 |  | *HV** |
| 47-A | Bologna | 6,44 | 9,68 |  | HV0* |
| 47-B | Bologna | 5,91 | 5,15 |  | *HV0** |
| 48-A | Bologna | 6,44 | 10,24 |  | U5a1 |
| 48-B | Bologna | 6,37 | 12,57 |  | *U5a1* |
| 49-A | Bologna | 5,08 | 0,00 |  | U7 |
| 49-B | Bologna | 5,98 | 0,00 |  | *U7* |
| 50-A | Bologna | 5,53 | 28,35 |  | W |
| 50-B | Bologna | 4,70 | 33,26 |  | *W* |
| 51-A | Bologna | 3,81 | 9,35 |  | T2 |
| 51-B | Bologna | 3,59 | 9,59 |  | *T2* |
| 52-A | Bologna | 5,60 | 6,44 |  | T1a |
| 52-B | Bologna | 5,76 | 9,92 |  | *T1a* |
| 53-A | Bologna | 4,25 | 6,29 |  | H1 |
| 53-B | Bologna | 3,44 | 7,69 |  | *H1* |
| 54-A | Bologna | 4,55 | 9,68 |  | K |
| 54-B | Bologna | 1,20 | 7,76 |  | *K* |
| 55-A | Bologna | 5,00 | 3,81 |  | N1b |
| 55-B | Bologna | 5,15 | 5,91 |  | *N1b* |
| 56-A | Bologna | 5,15 | 7,84 |  | H* |
| 56-B | Bologna | 4,92 | 15,44 |  | *H** |
| 57-A | Bologna | 8,71 | 6,68 |  | U6a |
| 57-B | Bologna | 6,60 | 0,00 |  | *U6a* |
| 58-A | Bologna | 6,14 | 5,38 |  | U4a |
| 58-B | Bologna | 5,76 | 41,63 |  | *U4a* |
| 59-A | Bologna | 4,18 | 0,00 |  | H* |
| 59-B | Bologna | 1,56 | 0,00 |  | *H** |
| 60-A | Bologna | 5,76 | 0,00 |  | J1c |
| 60-B | Bologna | 2,64 | 0,00 |  | *J1c* |
| 61-A | Bologna | 13,00 | 7,37 |  | K1 |
| 61-B | Bologna | 13,09 | 0,00 |  | *K1* |
| 62-A | Bologna | 5,68 | 7,84 |  | H* |
| 62-B | Bologna | 5,30 | 0,00 |  | *H** |
| 63-A | Bologna | 5,83 | 4,77 |  | U4 |
| 63-B | Bologna | 5,83 | 43,41 |  | *U4* |
| 64-A | Bologna | 5,08 | 8,39 |  | HV0a |
| 64-B | Bologna | 5,08 | 21,18 |  | *HV0a* |
| 65-A | Bologna | 4,77 | 11,56 |  | H1 |
| 65-B | Bologna | 5,30 | 15,00 |  | *H1* |
| 66-A | Bologna | 5,45 | 11,15 |  | H6 |
| 66-B | Bologna | 5,45 | 48,87 |  | *H6* |
| 67-A | Calabria | 9,43 | 5,08 |  | K1a4a1 |
| 67-B | Calabria | 11,15 | 4,48 |  | *K1a4a1* |
| 68-A | Calabria | 5,30 | 11,65 |  | H1a |
| 68-B | Calabria | 4,77 | 5,45 |  | *H1a* |
| 69-A | Calabria | 3,37 | 7,14 |  | H1 |
| 69-B | Calabria | 1,34 | 2,49 |  | *H1* |
| 70-A | Calabria | 0,84 | 1,70 |  | M1a |
| 70-B | Calabria | 12,91 | 15,71 |  | *M1a* |
| 71-A | Calabria | 4,33 | 0,91 |  | H8 |
| 71-B | Calabria | 5,08 | 1,84 |  | *H8* |
| 72-A | Calabria | 10,65 | 3,73 |  | H9 |
| 72-B | Calabria | 1,41 | 10,90 |  | *H9* |
| 73-A | Calabria | 4,77 | 5,68 |  | K1a |
| 73-B | Calabria | 3,07 | 6,60 |  | *K1a* |
| 74-A | Calabria | 4,33 | 5,53 |  | H5 |
| 74-B | Calabria | 5,53 | 5,91 |  | *H5* |
| 75-A | Calabria | 5,38 | 5,23 |  | J1 |
| 75-B | Calabria | 6,21 | 5,23 |  | *J1* |
| 76-A | Calabria | 3,51 | 3,73 |  | U6a1 |
| 76-B | Calabria | 2,78 | 22,39 |  | *U6a1* |
| 77-A | Calabria | 6,06 | 4,63 |  | X2 |
| 77-B | Calabria | 3,81 | 4,48 |  | *X2* |
| 78-A | Calabria | 4,70 | 10,16 |  | H* |
| 78-B | Calabria | 5,15 | 10,33 |  | *H** |
| 79-A | Calabria | 4,85 | 6,14 |  | H1b |
| 79-B | Calabria | 8,47 | 7,22 |  | *H1b* |
| 80-A | Calabria | 5,15 | 5,76 |  | H13a1a |
| 80-B | Calabria | 2,71 | 6,68 |  | *H13a1a* |
| 81-A | Calabria | 6,91 | 10,24 |  | K |
| 81-B | Calabria | 4,85 | 3,29 |  | *K* |
| 82-A | Calabria | 1,84 | 4,18 |  | J1c1 |
| 82-B | Calabria | 0,00 | 1,56 |  | *J1c1* |
| 83-A | Calabria | 0,00 | 3,96 |  | K1a |
| 83-B | Calabria | 3,29 | 3,37 |  | *K1a* |
| 84-A | Calabria | 6,29 | 5,08 |  | U6a1 |
| 84-B | Calabria | 5,08 | 5,68 |  | *U6a1* |
| 85-A | Calabria | 6,52 | 5,91 |  | H14 |
| 85-B | Calabria | 4,85 | 5,68 |  | *H14* |
| 86-A | Calabria | 6,91 | 6,14 |  | H* |
| 86-B | Calabria | 9,19 | 7,45 |  | *H** |
| 87-A | Calabria | 5,53 | 5,15 |  | X |
| 87-B | Calabria | 24,36 | 36,68 |  | *X* |
| 88-A | Calabria | 5,15 | 5,38 |  | N1b |
| 88-B | Calabria | 5,30 | 6,60 |  | *N1b* |
| 89-A | Calabria | 4,77 | 4,10 |  | H* |
| 89-B | Calabria | 2,35 | 3,29 |  | *H** |
| 90-A | Calabria | 7,30 | 3,44 |  | H* |
| 90-B | Calabria | 13,34 | 4,03 |  | *H** |
| 91-A | Calabria | 15,18 | 8,24 |  | N1a |
| 91-B | Calabria | 19,42 | 15,18 |  | *N1a* |
| 92-A | Calabria | 4,85 | 3,66 |  | H1 |
| 92-B | Calabria | 5,23 | 4,33 |  | *H1* |
| 93-A | Calabria | 10,90 | 11,40 |  | H* |
| 93-B | Calabria | 7,06 | 3,51 |  | *H** |
| 94-A | Calabria | 1,77 | 0,00 |  | T2b |
| 94-B | Calabria | 3,96 | 5,00 |  | *T2b* |
| 95-A | Calabria | 4,03 | 3,96 |  | H* |
| 95-B | Calabria | 3,66 | 11,98 |  | *H** |
| 96-A | Calabria | 4,77 | 11,48 |  | H* |
| 96-B | Calabria | 4,33 | 4,70 |  | *H** |
| 97-A | Calabria | 0,00 | 1,99 |  | H* |
| 97-B | Calabria | 10,00 | 3,15 |  | *H** |
| 98-A | Calabria | 7,06 | 6,37 |  | K1 |
| 98-B | Calabria | 4,25 | 4,77 |  | *K1* |
| 99-A | Calabria | 3,96 | 10,57 |  | HV* |
| 99-B | Calabria | 11,23 | 9,19 |  | *HV** |
| 100-A | Calabria | 6,60 | 8,32 |  | U1 |
| 100-B | Calabria | 6,60 | 18,38 |  | *U1* |
| 101-A | Calabria | 7,53 | 6,14 |  | H* |
| 101-B | Calabria | 7,30 | 6,98 |  | *H** |
| 102-A | Calabria | 5,83 | 4,55 |  | H* |
| 102-B | Calabria | 2,13 | 9,92 |  | *H** |
| 103-A | Calabria | 5,38 | 4,63 |  | HV0* |
| 103-B | Calabria | 6,60 | 11,40 |  | *HV0** |
| 104-A | Calabria | 5,60 | 5,23 |  | K1a |
| 104-B | Calabria | 6,98 | 7,22 |  | *K1a* |
| 105-A | Calabria | 6,21 | 7,22 |  | H* |
| 105-B | Calabria | 4,70 | 4,40 |  | *H** |
| 106-A | Calabria | 0,56 | 11,15 |  | HV0* |
| 106-B | Calabria | 10,82 | 8,47 |  | *HV0** |
| 107-A | Calabria | 3,37 | 5,53 |  | H* |
| 107-B | Calabria | 3,00 | 3,96 |  | *H** |
| 108-A | Calabria | 6,21 | 4,40 |  | K1 |
| 108-B | Calabria | 11,23 | 5,23 |  | *K1* |
| 109-A | Calabria | 5,53 | 3,44 |  | U9 |
| 109-B | Calabria | 5,30 | 9,51 |  | *U9* |
| 110-A | Calabria | 7,37 | 5,53 |  | HV* |
| 110-B | Calabria | 6,75 | 7,84 |  | *HV** |
| 111-A | Calabria | 10,57 | 11,07 |  | H* |
| 111-B | Calabria | 4,48 | 4,77 |  | *H** |
| 112-A | Calabria | 5,30 | 5,83 |  | U5a1 |
| 112-B | Calabria | 6,21 | 4,85 |  | *U5a1* |
| 113-A | Calabria | 5,08 | 6,91 |  | T2 |
| 113-B | Calabria | 4,77 | 5,45 |  | *T2* |
| 114-A | Calabria | 4,85 | 10,90 |  | T1a |
| 114-B | Calabria | 5,45 | 11,40 |  | *T1a* |
| 115-A | Calabria | 3,00 | 4,48 |  | J1c |
| 115-B | Calabria | 4,77 | 4,55 |  | *J1c* |
| 116-A | Calabria | 5,83 | 12,40 |  | H* |
| 116-B | Calabria | 2,49 | 11,73 |  | *H** |
| 117-A | Calabria | 6,29 | 2,71 |  | H* |
| 117-B | Calabria | 3,29 | 0,00 |  | *H** |
| 118-A | Calabria | 6,52 | 7,14 |  | H* |
| 118-B | Calabria | 4,77 | 6,98 |  | *H** |
| 119-A | Calabria | 6,06 | 6,14 |  | H* |
| 119-B | Calabria | 13,17 | 5,23 |  | H* |
| 120-A | Calabria | 3,37 | 4,55 |  | H2a1 |
| 120-B | Calabria | 10,90 | 2,42 |  | *H2a1* |
| 121-A | Calabria | 5,23 | 6,52 |  | H1 |
| 121-B | Calabria | 5,08 | 5,53 |  | *H1* |
| 122-A | Finland |  |  | 4,48 | H1a |
| 122-B | Finland |  |  | 4,77 | *H1a* |
| 123-A | Finland |  |  | 9,76 | U5b* |
| 123-B | Finland |  |  | 1,99 | *U5b** |
| 124-A | Finland |  |  | 2,49 | U2 |
| 124-B | Finland |  |  | 6,29 | *U2* |
| 125-A | Finland |  |  | 4,40 | U5b* |
| 125-B | Finland |  |  | 4,92 | *U5b** |
| 126-A | Finland |  |  | 5,53 | J2a* |
| 126-B | Finland |  |  | 5,23 | *J2a** |
| 127-A | Finland |  |  | 0,00 | U8a |
| 127-B | Finland |  |  | 0,00 | *U8a* |
| 128-A | Finland |  |  | 4,77 | H1 |
| 128-B | Finland |  |  | 0,00 | *H1* |
| 129-A | Finland |  |  | 5,23 | U5b* |
| 129-B | Finland |  |  | 5,15 | *U5b** |
| 130-A | Finland |  |  | 9,76 | I |
| 130-B | Finland |  |  | 2,35 | *I* |
| 131-A | Finland |  |  | 5,60 | U |
| 131-B | Finland |  |  | 0,00 | *U* |
| 132-A | Finland |  |  | 5,15 | H2 |
| 132-B | Finland |  |  | 4,40 | *H2* |
| 133-A | Finland |  |  | 3,51 | H3 |
| 133-B | Finland |  |  | 8,95 | *H3* |
| 134-A | Finland |  |  | 3,88 | X |
| 134-B | Finland |  |  | 5,38 | *X* |
| 135-A | Finland |  |  | 5,15 | D5a |
| 135-B | Finland |  |  | 4,63 | *D5a* |
| 136-A | Finland |  |  | 7,76 | H* |
| 136-B | Finland |  |  | 5,08 | *H** |
| 137-A | Finland |  |  | 5,00 | H1 |
| 137-B | Finland |  |  | 5,45 | *H1* |
| 138-A | Finland |  |  | 5,60 | J1c1 |
| 138-B | Finland |  |  | 5,45 | *J1c1* |
| 139-A | Finland |  |  | 6,75 | W |
| 139-B | Finland |  |  | 5,98 | *W* |
| 140-A | Finland |  |  | 0,00 | K2 |
| 140-B | Finland |  |  | 0,00 | *K2* |
| 141-A | Finland |  |  | 0,00 | H* |
| 141-B | Finland |  |  | 0,00 | *H** |
| 142-A | Finland |  |  | 0,00 | H1 |
| 142-B | Finland |  |  | 0,00 | *H1* |
| 143-A | Finland |  |  | 0,00 | H1a2 |
| 143-B | Finland |  |  | 5,53 | *H1a2* |
| 144-A | Finland |  |  | 5,30 | J1 |
| 144-B | Finland |  |  | 5,60 | J1 |
| 145-A | Finland |  |  | 5,68 | U5b* |
| 145-B | Finland |  |  | 5,91 | *U5b** |
| 146-A | Finland |  |  | 0,00 | H6 |
| 146-B | Finland |  |  | 0,00 | *H6* |
| 147-A | Finland |  |  | 2,85 | H1 |
| 147-B | Finland |  |  | 0,00 | *H1* |
| 148-A | Finland |  |  | 6,52 | W1 |
| 148-B | Finland |  |  | 6,98 | *W1* |
| 149-A | Finland |  |  | 4,70 | X |
| 149-B | Finland |  |  | 0,00 | *X* |
| 150-A | Finland |  |  | 0,00 | H1 |
| 150-B | Finland |  |  | 7,45 | *H1* |
| 151-A | Finland |  |  | 10,08 | H4 |
| 151-B | Finland |  |  | 0,00 | *H4* |
| 152-A | Finland |  |  | 6,44 | H1a2 |
| 152-B | Finland |  |  | 0,00 | *H1a2* |
| 153-A | Finland |  |  | 8,00 | T2b |
| 153-B | Finland |  |  | 3,81 | *T2b* |
| 154-A | Finland |  |  | 4,63 | U5a* |
| 154-B | Finland |  |  | 7,84 | *U5a** |
| 155-A | Finland |  |  | 4,25 | W |
| 155-B | Finland |  |  | 5,30 | *W* |
| 156-A | Finland |  |  | 5,15 | W |
| 156-B | Finland |  |  | 5,98 | *W* |
| 157-A | Finland |  |  | 6,21 | V |
| 157-B | Finland |  |  | 6,29 | *V* |
| 158-A | Finland |  |  | 5,68 | U5b* |
| 158-B | Finland |  |  | 5,38 | *U5b** |
| 159-A | Finland |  |  | 5,38 | H2 |
| 159-B | Finland |  |  | 5,76 | *H2* |
| 160-A | Finland |  |  | 5,53 | W |
| 160-B | Finland |  |  | 4,77 | *W* |
| 161-A | Finland |  |  | 0,00 | T2b |
| 161-B | Finland |  |  | 3,81 | *T2b* |
| 162-A | Finland |  |  | 7,84 | W4 |
| 162-B | Finland |  |  | 9,68 | *W4* |
| 163-A | Finland |  |  | 4,10 | H* |
| 163-B | Finland |  |  | 4,92 | *H** |
| 164-A | Finland |  |  | 0,00 | H2 |
| 164-B | Finland |  |  | 0,49 | *H2* |
| 165-A | Finland |  |  | 7,37 | H* |
| 165-B | Finland |  |  | 11,90 | *H** |
| 166-A | Finland |  |  | 6,52 | H7 |
| 166-B | Finland |  |  | 4,55 | *H7* |
| 167-A | Finland |  |  | 5,83 | U5b* |
| 167-B | Finland |  |  | 5,45 | *U5b** |
| 168-A | Finland |  |  | 2,42 | I |
| 168-B | Finland |  |  | 2,71 | *I* |
| 169-A | Finland |  |  | 0,00 | K1a |
| 169-B | Finland |  |  | 5,53 | *K1a* |
| 170-A | Finland |  |  | 9,35 | H4a1a1 |
| 170-B | Finland |  |  | 5,45 | *H4a1a1* |
| 171-A | Finland |  |  | 5,15 | H1 |
| 171-B | Finland |  |  | 6,68 | *H1* |
| 172-A | Finland |  |  | 3,88 | H1a2 |
| 172-B | Finland |  |  | 3,81 | *H1a2* |
| 173-A | Finland |  |  | 6,14 | H2 |
| 173-B | Finland |  |  | 0,00 | *H2* |
| 174-A | Finland |  |  | 5,60 | K2 |
| 174-B | Finland |  |  | 4,33 | *K2* |
| 175-A | Finland |  |  | 7,37 | H* |
| 175-B | Finland |  |  | 3,07 | *H** |
| 176-A | Finland |  |  | 6,44 | K1c |
| 176-B | Finland |  |  | 5,68 | *K1c* |
| 177-A | Finland |  |  | 5,76 | H2 |
| 177-B | Finland |  |  | 5,98 | *H2* |
| 178-A | Finland |  |  | 5,45 | H4a1a1 |
| 178-B | Finland |  |  | 5,60 | *H4a1a1* |
| 179-A | Finland |  |  | 6,60 | T1a |
| 179-B | Finland |  |  | 6,29 | *T1a* |
| 180-A | Finland |  |  | 6,37 | T |
| 180-B | Finland |  |  | 6,60 | *T* |
| 181-A | Finland |  |  | 7,84 | W |
| 181-B | Finland |  |  | 5,76 | *W* |
| 182-A | Finland |  |  | 5,45 | J1c* |
| 182-B | Finland |  |  | 5,38 | *J1c** |
| 183-A | Finland |  |  | 5,00 | V |
| 183-B | Finland |  |  | 5,08 | *V* |
| 184-A | Finland |  |  | 5,08 | T2 |
| 184-B | Finland |  |  | 9,35 | *T2* |
| 185-A | Finland |  |  | 10,24 | H1a2 |
| 185-B | Finland |  |  | 9,68 | *H1a2* |
| 186-A | Finland |  |  | 6,44 | U5b* |
| 186-B | Finland |  |  | 5,76 | *U5b** |
